# Management and outcome of mesh infection after abdominal wall reconstruction in a tertiary care center

**DOI:** 10.1007/s10029-025-03265-3

**Published:** 2025-01-23

**Authors:** Victor Franchi, Claire Triffault-Fillit, Sophie Jarraud, Jean-Yves Mabrut, Clément Javaux, Olivier Monneuse, Anne Conrad, Tristan Ferry, Maud Robert, Florence Ader, Guillaume Passot, Florent Valour

**Affiliations:** 1 Department of Infectious Diseases, Hospices Civils de Lyon, Service des Maladies Infectieuses et Tropicales, 103 Grande Rue de la Croix-Rousse, Lyon, 69004 France; 2 Laboratory of Bacteriology, Institut des Agents Infectieux, Hospices Civils de Lyon, Lyon, France; 3 International Center for Research in Infectiology (CIRI), Inserm, U1111, Université Claude Bernard Lyon 1, CNRS, UMR5308, Ecole Normale Supérieure de Lyon, Univ Lyon, Lyon, France; 4 Department of General Surgery and Liver Transplantation, Croix Rousse University Hospital, Hospices Civils de Lyon, Lyon, France; 5 Institute of Hepatology Lyon (IHL), INSERM U1052, Lyon, France; 6 Emergency and General Surgery Unit, Edouard Herriot Hospital, Hospices Civils de Lyon, Lyon, France; 7 UMR CNRS 5558, Laboratoire de Biométrie et Biologie Évolutive, Univ Lyon, Université Claude Bernard Lyon 1, Villeurbanne, France; 8 Department of Digestive and Bariatric Surgery, Edouard Herriot Hospital, Hospices Civils de Lyon, Lyon, France; 9 CarMeN Laboratory, INSERM U1060, Lyon 1 University, Lyon, France; 10 Department of General Surgery and Surgical Oncology, Lyon-Sud Hospital, Hospices Civils de Lyon, Pierre-Bénite, France; 11 CICLY, Lyon 1 University, Lyon, France

**Keywords:** Abdominal wall repair, Hernia mesh infection, Microbiology, Treatment outcomes, Conservative treatment

## Abstract

**Purpose:**

Abdominal wall reconstruction is a common surgical procedure, with a post-operative risk of mesh-associated infection of which management is poorly known. This study aims to comprehensively analyze clinical and microbiological aspects of mesh infection, treatment modalities, and associated outcomes.

**Methods:**

Patients with abdominal mesh infection were included in a retrospective observational cohort (2010-2023). Patients characteristics and management were described, and determinants for failure were assessed by logistic regression and treatment failure-free survival curve analysis (Kaplan-Meier).

**Results:**

Two hundred and nine patients (median age, 62 [IQR, 55-71] years) presented a mesh infection occurring within 15 (IQR, 7-31) days after surgery, mainly as an abdominal wall or deep abscess (*n*=189, 90.4%). Infection was polymicrobial in 89/166 (79.4%) cases, *S. aureus* (*n*=60, 36.1%), *Enterobacteriaceae* (*n*=60, 36.1%) and anaerobes (*n*=40, 24.1%) being the most prevalent pathogens. Surgery was performed in 130 (62.2%) patients, associated with a 13.5 (IQR, 8-21) day course of antimicrobial therapy in 172/207 (83.1%) cases. Sixty-three (30.1%) treatment failures occurred, associated with previous multiple abdominal surgeries (OR, 3.305; 95%CI, 1.297-8.425), complete mesh removal (OR, 0.145; 95%CI, 0.063-0.335) and antimicrobial therapy (OR, 0.328; 95%CI, 0.136-0.787). The higher failure rate of conservative strategies was associated with symptom duration >1 month (OR, 3.378; 95%CI, 1.089-4.005) and retromuscular mesh position (OR, 0.444; 95%CI, 0.199-0.992).

**Conclusion:**

Mesh infection is associated with high treatment failure rates. Complete mesh removal coupled with targeted antibiotic therapy is associated with better outcomes. Conservative treatment strategies must rely on careful patient selection based on symptom duration and mesh placement.

## Introduction

Reconstruction of abdominal wall defect can be challenging, especially in comorbid patients and/or with complex hernias, following multiple surgeries or abdominal trauma. The final strategy must be safe and effective, and the use of mesh is now one of the most widely-performed surgical procedures [1, 2]. Post-operative infection represents a notable complication, with reported incidences ranging from 0.3% in clean inguinal hernia repairs to as high as 19.6% in complicated hernia cases [3, 4]. Such infections can lead to severe consequences, including sepsis, need for subsequent surgeries, development of chronic fistulas, persistent pain, or hernia recurrence [5]. Determinants of post-operative mesh infection have been extensively documented in existing literature [3, 6, 7]. They include mainly patient- and surgery-related parameters, such as age, body mass index (BMI), smoking status, length of surgery, open surgery compared with laparoscopic procedures, and hernia localization.

Various management strategies have been described [8–10], involving the following interventions, alone or in combination: systemic antibiotic therapy, percutaneous drainage, complete or partial mesh removal, and negative pressure therapy [11]. However, these strategies have been poorly investigated, and criteria guiding clinicians in the selection of the most appropriate therapeutic approach are not available [12].

The objective of this study is to provide a comprehensive account of the clinical and microbiologic aspects of mesh infection observed in our tertiary care hospital, while describing treatment modalities employed and associated outcomes.

## Patients and methods

### Ethical statement

The study received the approval of the Scientific and Ethical Committees of Hospices Civils de Lyon, France (reference number 23-5457). In accordance with French legislation regarding retrospective observational studies, all patients received written information about the study and their possibility to decline to participate, but the need for written informed consent was waived.

### Study design and data collection

Patients diagnosed with abdominal mesh infection in the four digestive surgery wards of our tertiary care center were identified from records from 2010 to 2023 and included in a retrospective observational cohort study. Patients were identified through electronic medical records by cross-referencing medical documents with keywords related to mesh infection, and using diagnosis data associated with each hospital stay, including a combination of International Classification of Diseases (ICD) codes, and hospital pharmacy registries regarding mesh placement and removal. In patients having experienced several episodes of mesh-related infections, only the first episode was considered.

For each patient, data were collected from medical records into an anonymous standardized case report form. Co-morbidities were summarized using the modified Charlson comorbidity index [13], and the American Society of Anesthesiologists (ASA) score.

### Definitions

The diagnosis of mesh infection was based on clinical evaluation by the treating surgeon. Infections involving skin and subcutaneous tissue only were categorized as superficial. All other infections were considered as deep. Assessment of the depth of infection was performed by collecting clinical and surgical data, and imaging reports when available. Patients with the superficial wounds who did not undergo subsequent imaging nor surgery were classified as superficial infection on the basis of clinical findings, only. The time to infection was calculated as the number of days between mesh placement or the last surgery at the site and the onset of symptoms. Antimicrobial therapy was deemed appropriate when all pathogens identified in gold-standard sample cultures were targeted by at least one antimicrobial agent. Treatment was considered conservative in every case where the initial management strategy did not involve complete removal of the mesh. Treatment failure was defined as infection persistence or relapse after treatment on a clinical basis (i.e., wound abnormalities suspected of infection, abscess and/or fistula) and/or the need for additional surgery motivated by septic reasons and not planned in the initial treatment scheme.

Microbiology etiology was described according to the results of cultures from normally sterile site samples, excluding superficial swab. Common contaminants, such as Coagulase-Negative *Staphylococci* (CNS), *Corynebacterium* spp. or *Cutibacterium* spp., were considered only when found in at least two samples.

The mesh material was described according to the manufacturer’s notice, describing both the material used (synthetic, semi-synthetic, biologic) and its resorption characteristics once implanted (absorbable, semi-absorbable, non-absorbable).

### Statistical analysis

Variables were presented as percentages for dichotomous variables and as medians with interquartile ranges (IQR) for continuous variables. In percentage calculations, missing values were excluded from the denominator. Groups were compared using non-parametric tests (Chi-square, Fisher exact, and Mann-Whitney U tests), as appropriate. Kaplan-Meier curves were used to compare treatment-failure free survival rates between groups, with statistical significance assessed by the log-rank (Mantel-Cox) test. The same analysis process was applied to the subgroup of patients who did not undergo complete mesh removal (conservative treatment group). After analysis of the whole cohort, and in order to consider a potential recruitment bias, patients primarily managed in our reference center (HCL group), i.e. who were not referred due to a previous mesh infection management failure, were independently analyzed.

Determinants of treatment failure were examined using stepwise binary logistic regression and expressed as odds ratios (ORs) along with their corresponding 95% confidence intervals (95%CI). Variables included in the final multivariate model were selected from non-interacting variables with clinical significance and *p*-values obtained in univariate analysis less than 0.15. A *p*-value of less than 0.05 was considered statistically significant. All statistical analyses were performed using SPSS version 19.0 (SPSS, Chicago, IL, USA) and GraphPad Prism version 5.03 (GraphPad, San Diego, CA, USA) softwares.

## Results

### Included population and index surgery

A total of 209 patients were included, comprising 119 (56.9%) males with a median age of 62 (IQR, 55–71) years. The median modified Charlson’s comorbidity Index was 3 (IQR, 2–3), main comorbidities being obesity (median body mass index [BMI], 29.7 [IQR, 26.4–33.2]; >30 in 96 [46.8%] patients), diabetes mellitus (*n* = 60, 28.7%) and solid tumor or hemopathy (*n* = 55, 26.3%). Patient and infection characteristics are summarized in Table 1. The majority (*n* = 199, 95.2%) of patients had undergone previous abdominal surgery, with multiple procedures in 166 (79.4%) cases. Abdominal wall repair was planned for incisional hernias in 154 (76.2%) patients, mostly using synthetic (*n* = 131/175, 74.9%) and non-absorbable (*n* = 83/173, 50.9%) meshes. The most frequent mesh locations were retromuscular (76/190, 40.0%) and intraperitoneal (56/190, 29.5%). Antibiotics were administered as prophylaxis during surgery to 109/122 (89.3%) patients, with cefazolin being the most frequently used drug.

**Table 1:** Description of the included population, comparison of patients without or with treatment failure, and determinants of treatment failure (univariate analysis)

				All patients	Descriptive analysis			Univariate analysis	
					Failure	Success	*p*-value	OR (95%CI)	*p*-value
**n**				209	63	146			
**Demographics and comorbidities**									
	Gender (male)			119/209 (56.9%)	38/63 (60.3%)	81/146 (55.5%)	0.517	1.220 (0.669–2.225)	0.517
	Age (years)			62 (55–71)	61 (55-69.5)	63 (55–72)	0.432	0.992 (0.969–1.016)	0.528
	BMI (kg/m²)			29.7 (26.4–33.2)	29.9 (27.3–32.9)	29.7 (26.1–33.3)	0.606	1. 012 (0.963–1.063)	0.634
	ASA score			2 (2–3)	2 (2-2.3)	2 (2–3)		0.793 (0.490–1.281)	0.342
	Modified Charlson’s comorbidity index			3 (2–5)	3 (1.5-4)	3 (2–5)		0.972 (0.857–1.103)	0.663
	Active smoking			42/209 (20.1%)	14/63 (22.2%)	28/146 (19.2%)	0.614	1.204 (0.584–2.481)	0.615
	Previous abdominal surgery			199/209 (95.2%)	59/63 (93.7%)	140/146 (95.9%)	0.486	0.632 (0.172–2.322)	0.490
		> 1 previous abdominal surgeries		166/209 (79.4%)	56/63 (88.9%)	110/146 (75.3%)	0.026	2.618 (1.096–6.257)	0.030
**Index surgery**									
	Operative indication								
		Primary hernia		42/202 (20.8%)	14/62 (22.6%)	28/140 (20.0%)	0.677	1.167 (0.565–2.409)	0.677
		Incisional hernia		154/202 (76.2%)	45/62 (72.6%)	109/140 (77.9%)	0.416	0.753 (0.379–1.495)	0.417
		Acute complication		11/202 (5.4%)	4/62 (6.5%)	7/140 (5.0%)	0.740	1.310 (0.369–4.650)	0.676
		Post-surgical parietal closure		6/202 (3.0%)	3/62 (4.8%)	3/140 (2.1%)	0.374	2.322 (0.455–11.841)	0.311
	Mesh characteristics								
		Size (cm²)		400 (225-775.5)	500 (300–900)	360 (225-667.5)	0.106	1.001 (1.000-1.002)	0.058
		Synthetic		131/175 (74.9%)	41/58 (70.7%)	90/117 (76.9%)	0.371	0.724 (0.356–1.472)	0.372
		Biosynthetic		43/175 (24.6%)	17/58 (29.3%)	26/117 (22.2%)	0.305	1.451 (0.711–2.963)	0.307
		Biological		1/175 (0.6%)	0/58 (0%)	1/117 (0.9%)	1.000	0.991 (0.975–1.008)	
		Absorbable		34/173 (19.7%)	15/57 (26.3%)	19/116 (16.4%)	0.122	1.823 (0.846–3.929)	0.125
		Semi-absorbable		51/173 (29.5%)	13/57 (22.8%)	38/116 (32.8%)	0.177	0.606 (0.292–1.259)	0.179
		Non-absorbable		88/173 (50.9%)	29/57 (50.9%)	59/116 (50.9%)	0.999	1.001 (0.531–1.887)	0.999
	Mesh location								
		Subcutaneous		37/190 (19.5%)	11/60 (18.3%)	26/130 (20.0%)	0.787	0.898 (0.411–1.964)	0.787
		Retromuscular		76/190 (40.0%)	23/60 (38.3%)	53/130 (40.8%)	0.750	0.903 (0.482–1.691)	0.750
		Preperitoneal		24/190 (12.6%)	6/60 (10.0%)	18/130 (13.8%)	0.458	0.691 (0.260–1.841)	0.460
		Intraperitoneal		56/190 (29.5%)	22/60 (36.7%)	34/130 (26.2%)	0.140	1.635 (0.849–3.146)	0.141
	Altemeier class 1			165/190 (86.8%)	51/60 (85%)	114/130 (87.7%)	0.610	0.795 (0.330–1.919)	0.610
**Infection presentation**									
	Time from surgery to onset of symptoms (days)			15 (7–31)	15 (7.3–31)	17 (7–31)	0.662	0.999 (0.999-1.000)	0.110
	Highest CRP level (mg/L)			124 (50–200)	130 (60–250)	114 (40.8-183.5)	0.238	1.002 (0.999–1.005)	0.179
	Infection type								
		Inflammatory wound dehiscence		9/209 (4.3%)	0/63 (0%)	9/146 (6.2%)	0.060	-	
		Abscess		189/209 (90.4%)	61/63 (96.8%)	128/146 (87.7%)	0.042	4.289 (0.964–19.076)	0.056
			Abdominal wall abscess	177/209 (84.7%)	56/63 (88.9%)	121/146 (82.9%)	0.268	1.653 (0.675–4.049)	0.272
			Deep abscess	25/209 (12.0%)	8/63 (12.7%)	17/146 (11.6%)	0.829	1.104 (0.450–2.708)	0.829
		Sinus tract		127/209 (60.8%)	42/63 (66.7%)	85/146 (58.2%)	0.251	1.435 (0.773–2.664)	0.252
			Enterocutaneous sinus tract	20/209 (9.6%)	4/63 (6.3%)	16/146 (11.0%)	0.443	0.551 (0.177–1.719)	0.304
		Early post-operative peritonitis		4/209 (1.9%)	1/63 (1.6%)	3/146 (2.1%)	1.000	0.769 (0.078–7.537)	0.821
**Microbiology results**				166/209 (79.4%)	51/63 (81.0%)	115/146 (78.8%)	0.720	1.146 (0.545–2.410)	0.720
	Sterile			10/166 (6.0%)	4/51 (7.8%)	6/115 (5.2%)	0.498	1.546 (0.417–5.733)	0.515
	Polymicrobial			89/166 (53.6%)	28/51 (54.9%)	61/115 (53.0%)	0.825	1.078 (0.556–2.089)	0.825
	*S.aureus*			60/166 (36.1%)	17/51 (33.3%)	43/115 (37.4%)	0.616	0.837 (0.418–1.676)	0.616
	CoNS			11/166 (6.6%)	4/51 (7.8%)	7/115 (6.1%)	0.738	1.313 (0.367–4.701)	0.676
	*Streptococcus* spp.			21/166 (12.7%)	4/51 (7.8%)	17/115 (14.8%)	0.312	0.491 (0.156–1.539)	0.222
	*Enterococcus* spp.			33/166 (19.9%)	7/51 (13.7%)	26/115 (22.6%)	0.186	0.545 (0.219–1.352)	0.190
	*Corynebacterium* spp.			3/166 (1.8%)	2/51 (3.9%)	1/115 (0.9%)	0.224	4.653 (0.412–52.521)	0.214
	*Cutibacterium* spp			5/166 (3.0%)	3/51 (5.9%)	2/115 (1.7%)	0.170	3.531 (0.572–21.811)	0.174
	Enterobacteriaceae			60/166 (36.1%)	19/51 (37.3%)	41/115 (35.7%)	0.843	1.072 (0.541–2.124)	0.843
	*Pseudomonas* spp.			10/166 (6.0%)	3/51 (5.9%)	7/115 (6.1%)	1.000	0.964 (0.239–3.889)	0.959
	Anaerobes			40/166 (24.1%)	11/51 (21.6%)	29/115 (25.2%)	0.612	0.816 (0.371–1.795)	0.612
	*Actinomyces* spp.			9/166 (5.4%)	2/51 (3.9%)	7/115 (6.1%)	0.723	0.630 (0.126–3.142)	0.573
	Fungi			12/166 (7.2%)	4/51 (7.8%)	8/115 (7.0%)	0.533	1.138 (0.327–3.966)	0.839
**Infection management**									
	Local treatment only			46/209 (22.0%)	22/63 (34.9%)	24/146 (16.4%)	0.003	2.728 (1.384–5.374)	0.004
	Radiological drainage			19/209 (9.1%)	6/63 (9.5%)	13/146 (8.9%)	1.000	1.077 (0.390–2.974)	0.886
	Surgery			130/209 (62.2%)	29/63 (46%)	101/146 (69.2%)	0.002	0.380 (0.207–0.698)	0.002
		Complete mesh removal		78/209 (37.3%)	9/63 (14.3%)	69/146 (47.3%)	< 10^− 3^	0.186 (0.86 − 0.404)	< 10^− 3^
		Partial mesh removal		11/209 (5.3%)	3/63 (4.8%)	8/146 (5.5%)	1.000	0.863 (0.221–3.364)	0.831
		Single-stage abdominal wall reconstruction		16/129 (12.4%)	3/29 (10.3%)	13/100 (13.0%)	1.000	0.772 (0.204–2.919)	0.703
	Post-operative negative pressure therapy			38/209 (18.2%)	10/63 (15.9%)	28/146 (19.2%)	0.570	0.795 (0 0.360-1.755)	0.570
**Antibiotic treatment**				172/207 (83.1%)	47/62 (75.8%)	125/145 (86.2%)	0.067	0.501 (0.237–1.060)	0.071
	Appropriate empiric antibiotic therapy			99/131 (75.6%)	25/33 (75.8%)	74/98 (75.5%)	0.977	1.014 (0.404–2.542)	0.977
	Secondary adaptation			79/123 (64.2%)	15/29 (51.7%)	64/94 (68.1%)	0.108	0.502 (0.215–1.172)	0.111
	Treatment duration (days)			13.5 (8–21)	10 (7–19)	14 (9–21)	0.239	0.998 (0.975–1.021)	0.835
**Outcomes**									
	Failure			63/209 (30.1%)	63/63 (100%)	NA	NA	NA	NA
	Time to failure (days)			38 (16–183)	38 (16–183)	NA	NA	NA	NA
	Hernia recurrence			67/209 (32.1%)	20/63 (31.7%)	47/146 (32.2%)	0.949	0.980 (0.520–1.847)	0.949
	Subsequent surgery for infectious purpose			48/209 (23.0%)	48/63 (76.2%)	NA	NA	NA	NA
	Follow-up time (weeks)			73 (26.4-191.1)	74.9 (32.7-211.8)	72 (24.5-189.8)	0.316	1.001 (0.999–1.004)	0.199

95%CI, 95% confidence interval; ASA, American Society of Anesthesiologists, BMI, Body mass index; CoNS, Coagulase negative staphylococci; CRP, C-reactive protein; NA, Not applicable; OR, Odd ratio

Forty patients (19.1%) were referred to our center after initial management elsewhere. Of note, baseline characteristics of this subset of patients were not statistically different from patients primarily managed in our center (data not shown).

### Infection presentation

Median time between index surgery and symptoms was 15 (IQR, 7–31) days. Fever was present in 88 (42.1%) patients, only. A CT-scan was performed in 167 (76.9%) patients. Abdominal wall and deep abscesses were observed in 177 (84.7%) and 25 (12.0%) patients, respectively. Sinus tracts were identified in 127 (60.8%) patients. Less common presentations included enterocutaneous fistula (*n* = 20, 9.6%) and isolated inflammatory wound dehiscence (*n* = 9, 4.3%).

### Microbiology findings

Reliable samples for microbiological examination were obtained from 166 (79.4%) patients. Ten (6%) samples were culture-sterile. Cultures revealed polymicrobial infections in 86 (53.6%) cases. *Staphylococcus aureus* (*n* = 60, 36.1%) and *Enterobacteriaceae* (*n* = 60, 36.1%) were the most frequently isolated organisms. Multidrug-resistant (MDR) bacteria were uncommon, with 3/60 (5%) methicillin-resistant *S. aureus*, and 9/60 (15%) *Enterobacteriaceae* resistant to 3rd generation cephalosporins. Surgeries involving progressive preoperative pneumoperitoneum were associated with higher occurrence of MDR bacterial infection (3/13 *versus* 9/196, OR, 6.964 [1.509–32.142]; *p* = 0.013).

**Fig. 1:**
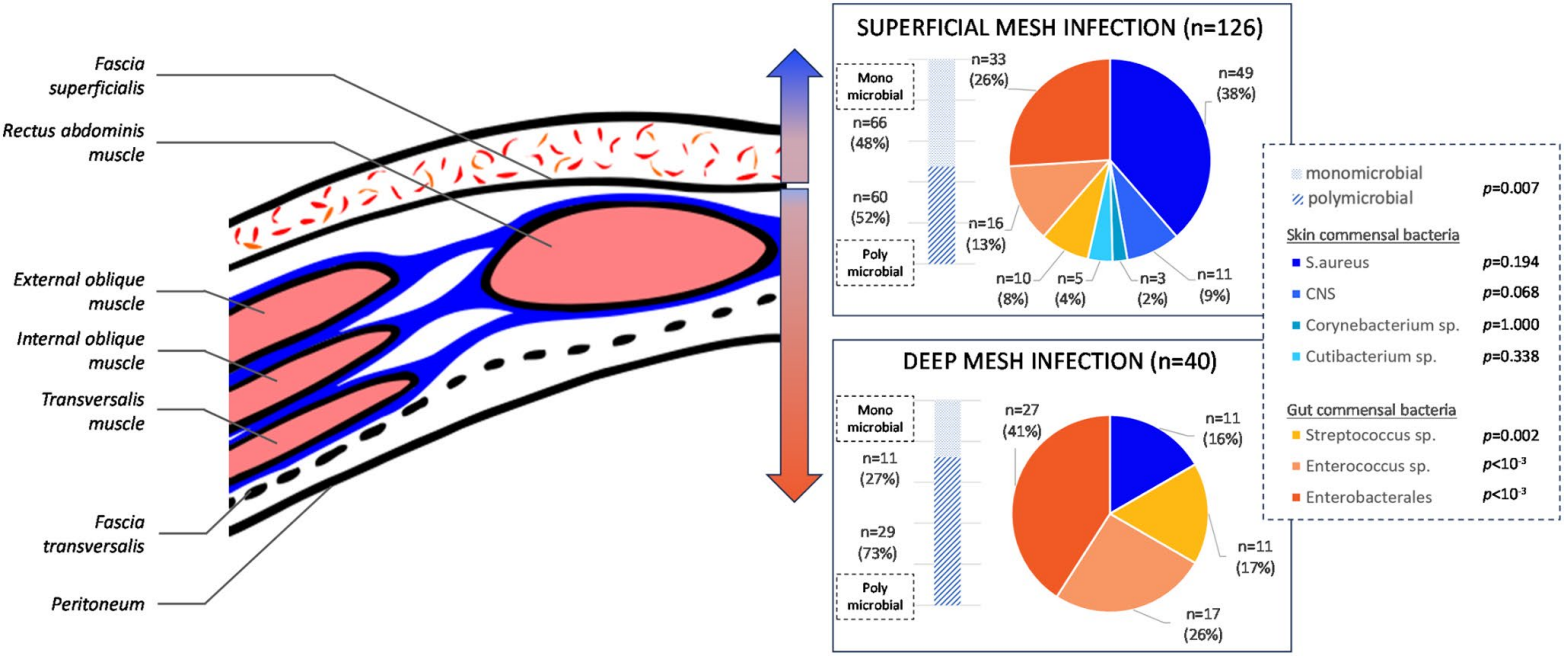
Microbial distribution according to infection site

Microbiology data highlights significant differences toward clinical presentations (Fig. 1).

### Infection management

Surgical intervention was undertaken in 130 (62.2%) cases, with total or partial mesh removal in 78 (37.3%) and 11 (5.3%) cases, respectively. Single stage abdominal wall reconstruction with a new mesh was performed in 16/129 (12.4%) patients, predominantly using absorbable meshes. Post-operative negative pressure therapy was utilized in 38 (18.2%) patients, while radiological drainage was performed in 19 (9.1%) patients. Antibiotic treatment was prescribed to 172/207 (83.1%) patients for a median duration of 13.5 (IQR, 8–21) days, with therapy deemed appropriate in 99/131 (75.6%) cases.

### Outcomes

**Fig. 2:**
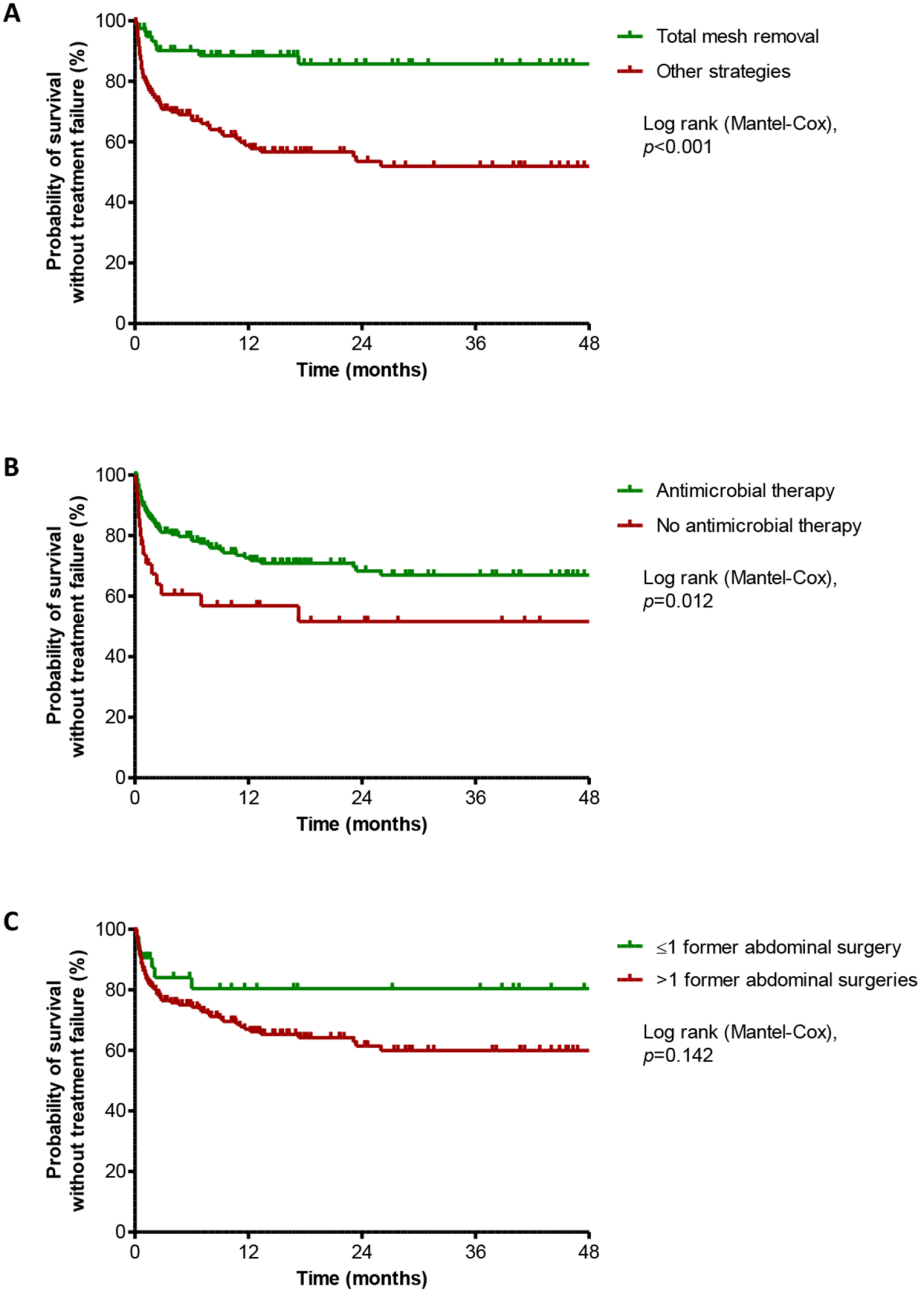
Kaplan-Meier survival curve for probability of survival without treatment failure in all patients with mesh infection after abdominal wall reconstruction, according to the main determinants of outcome

After a median follow-up of 73 (IQR, 26.4-191.1) weeks, treatment failure was observed in 63 (30.1%) patients, with a median time to failure of 38 (IQR, 16–183) days after the end of initial infection care. Most patients experiencing failure (*n* = 48/63, 76.2%) underwent subsequent surgery for infectious purposes. Baseline characteristics of patients with treatment failure and success were similar (Table 1). In univariate analysis, the only comorbidity associated with failure was multiple previous abdominal surgeries (OR, 2.618; 95%CI, 1.096–6.257; *p* = 0.030). Index surgery parameters, clinical presentation and implicated pathogens were not significantly related with outcomes. Surgical treatment (OR, 0.380; 95%CI, 0.207–0.698; *p* = 0.002), and especially total mesh removal (OR, 0.186; 95%CI, 0.860 − 0.404; *p* < 10^− 3^), were protective for treatment failure (Fig. 2). Of note, the evaluation of outcome predictors of the subset of patients primarily managed in our center did not allow to highlight specific risk factors compared to the whole population (data not shown).

In multivariate analysis, multiple previous abdominal surgeries (OR, 3.305; 95%CI, 1.297–8.425; *p* = 0.012), complete mesh removal (OR, 0.145; 95%CI, 0.063–0.335; *p* < 10^− 3^) and antibiotic therapy (OR, 0.328; 95%CI, 0.136–0.787; *p* = 0.013) were independent predictors of treatment outcome. Of note, outcome of patients referred to our center after a previous failure of infection management was similar.

### Conservative treatment group

**Fig. 3:**
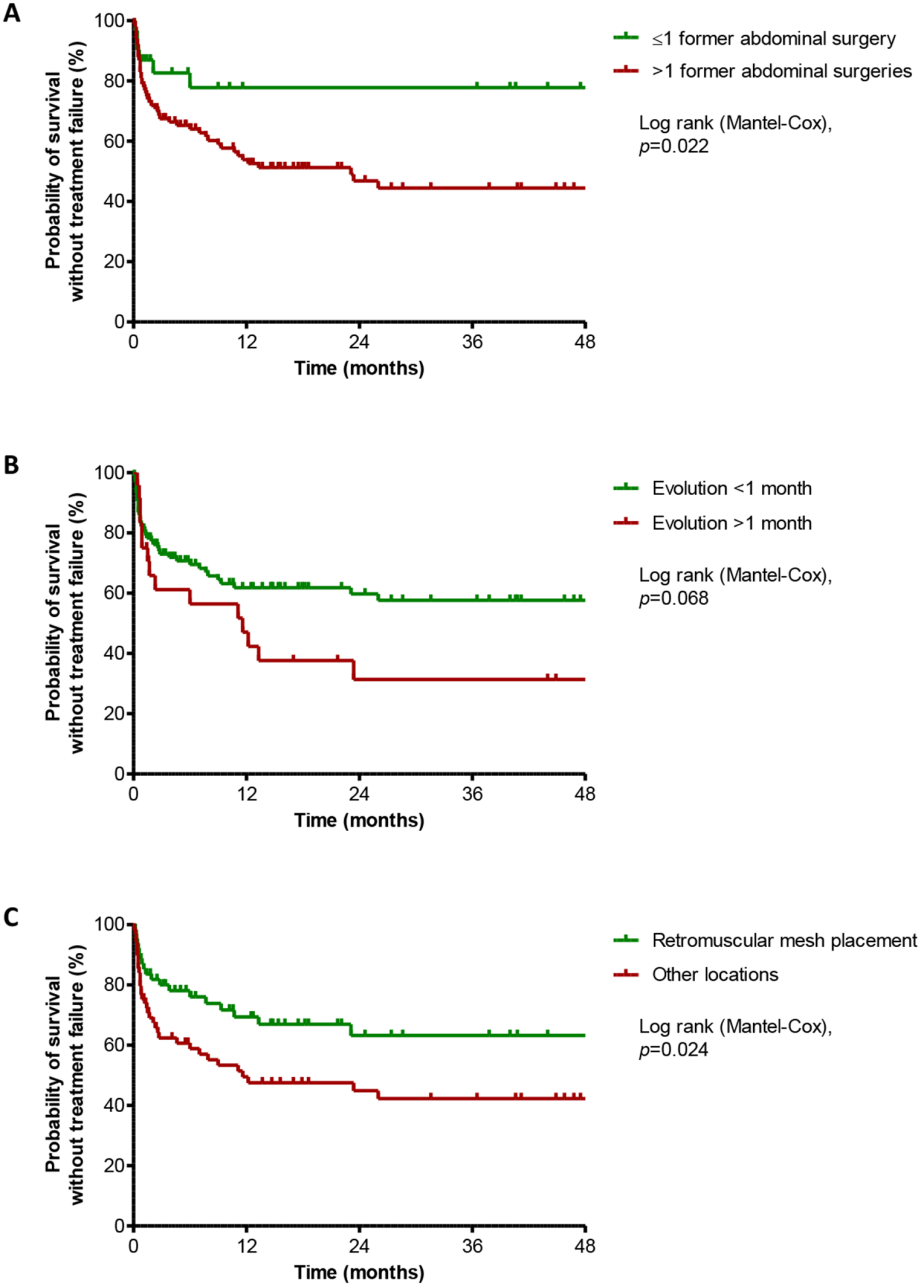
Kaplan-Meier survival curve for probability of survival without treatment failure after conservative management of patients with mesh infection after abdominal wall reconstruction, according to the main determinants of outcome

The 131 patients who underwent conservative strategy are presented in Table 2. After a median follow-up of 76 (IQR, 25.9-201.1) weeks, treatment failure was observed in 54 (41.2%) patients, with a median time to failure of 36.5 (IQR, 16–183) days after the end of initial infection care. In this subgroup, factors associated with treatment failure were multiple previous abdominal surgeries (OR, 3.846; 95%CI, 1.452–10.182; *p* = 0.006), intraperitoneal mesh placement (OR, 2.723; 95%CI, 1.216–6.096; *p* = 0.014), symptoms duration superior to one month (OR, 3.051; 95%CI, 1.175–7.919; *p* = 0.021), and presence of a fistula (OR, 2.162; 95%CI, 0.965–6.556; *p* = 0.036). Retromuscular mesh placement (OR, 0.428; 95%CI, 0.207–0.886; *p* = 0.022) and antibiotic treatment (OR, 0.381; 95%CI, 0.137–1.060; *p* = 0.064) emerged as protective factors against treatment failure (Fig. 3).

**Table 2:** Description of the conservative treatment subgroup, comparison of patients without or with treatment failure, and determinants of treatment failure (univariate analysis)

				Conservative treatment	Descriptive analysis			Univariate analysis	
					Failure	Success	*p*-value	OR (95% CI)	*p*-value
**n**				131	54	77			
**Demographics and comorbidities**									
	Gender (male)			74/131 (56.5%)	32/54 (59.3%)	42/77 (54.5%)	0.592	1.212 (0.599–2.451)	0.592
	Age (years)			62 (55–69)	61.5 (55.3–69.8)	62 (54.5–69)	0.924	1.006 (0.976–1.036)	0.681
	BMI (kg/m²)			29.7 (26.9–33.2)	29.1 (26.8–32.6)	30.1 (26-33.6)	0.680	0.993 (0.937–1.052)	0.825
	ASA score			2 (2–3)	2 (2–3)	2 (2–3)	0.825	0.965 (0.547–1.704)	0.904
	Modified Charlson’s Comorbidity index			3 (2-4.5)	3 (2–4)	3 (2–5)	0.945	1.003 (0.868–1.157)	0.966
	Active smoking			25/131 (19.1%)	11/54 (20.4%)	14/77 (18.2%)	0.754	1.151 (0.477–2.774)	0.753
	Previous abdominal surgery			126/131 (96.2%)	51/54 (94.4%)	75/77 (97.4%)	0.403	0.453 (0.073–2.809)	0.395
	> 1 previous abdominal surgeries			100/131 (76.3%)	48/54 (88.9%)	52/77 (67.5%)	0.005	3.846 (1.452–10.182)	0.006
**Index surgery**									
	Operative indication								
		Primary hernia		23/129 (17.8%)	11/53 (20.8%)	12/76 (15.8%)	0.469	1.396 (0.564–3.456)	0.469
		Incisional hernia		102/129 (79.1%)	39/53 (73.6%)	63/76 (82.9%)	0.201	0.574 (0.244–1.350)	0.203
		Acute complication		6/129 (4.7%)	3/53 (5.7%)	3/76 (3.9%)	0.689	1.459 (0.283–7.528)	0.651
		Post-surgical parietal closure		4/129 (3.1%)	3/53 (5.7%)	1/76 (1.3%)	0.305	4.499 (0.455–44.494)	0.198
	Mesh characteristics								
		Size (cm²)		500 (288–900)	500 (300–900)	450 (0-900)	0.301	1.000 (0.999–1.001)	0.178
		Synthetic		82/115 (71.3%)	32/49 (65.3%)	50/66 (75.8%)	0.220	0.602 (0.266–1.359)	0.222
		Biosynthetic		32/115 (27.8%)	17/49 (34.7%)	15/66 (22.7%)	0.157	1.806 (0.793–4.113)	0.159
		Biological		1/115 (0.9%)	0/49 (0%)	1/66 (1.5%)	1.000		
		Absorbable		29/116 (25%)	14/49 (28.6%)	15/67 (22.4%)	0.447	1.386 (0.595–3.228)	0.448
		Semi-absorbable		35/116 (30.2%)	11/49 (22.4%)	24/67 (35.8%)	0.121	0.518 (0.224–1.197)	0.123
		Non-absorbable		52/116 (44.8%)	24/49 (49.0%)	28/67 (41.8%)	0.442	1.337 (0.637–2.805)	0.442
	Mesh location								
		Subcutaneous		25/127 (19.7%)	11/52 (21.2%)	14/75 (18.7%)	0.729	1.168 (0.483–2.827)	0.729
		Retromuscular		62/127 (48.8%)	19/52 (36.5%)	43/75 (57.3%)	0.021	0.428 (0.207–0.886)	0.022
		Preperitoneal		8/127 (6.3%)	4/52 (7.7%)	4/75 (5.3%)	0.715	1.479 (0.352–6.202)	0.592
		Intraperitoneal		34/127 (26.8%)	20/52 (38.5%)	14/75 (18.7%)	0.013	2.723 (1.216–6.096)	0.014
	Altemeier class 1			110/128 (85.9%)	44/52 (84.6%)	66/76 (86.8%)	0.722	0.833 (0.305–2.276)	0.722
**Infection presentation**									
	Time from surgery to onset of symptoms (days)			14 (8–26)	13 (7–50)	14 (8-23.3)	0.801	1.000 (0.999-1.000)	0.533
	Highest CRP level (mg/L)			130 (60–205)	142 (60–250)	124.5 (0-180.8)	0.234	1.002 (0.998–1.005)	0.213
	Infection type								
		Inflammatory wound dehiscence		9/131 (6.9%)	0/54 (0%)	9/77 (11.7%)	0.009	NC	NC
		Abscess		120/131 (91.6%)	53/54 (98.1%)	67/77 (87.0%)	0.026	7.910 (0.981–63.761)	0.052
			Abdominal wall abscess	114/131 (87.0%)	49/54 (90.7%)	65/77 (84.4%)	0.289	1.809 (0.597–5.474)	0.293
			Deep abscess	13/131 (9.9%)	7/54 (13.0%)	6/77 (7.8%)	0.330	1.762 (0.557–5.571)	0.334
		Sinus tract		73/131 (55.7%)	36/54 (66.7%)	37/77 (48.1%)	0.035	2.162 (1.051–4.446)	0.036
			Enterocutaneous sinus tract	6/131 (4.6%)	4/54 (7.4%)	2/77 (2.6%)	0.229	2.999 (0.529–17.001)	0.214
		Early post-operative peritonitis		0/131 (0%)	0/54 (0%)	0/77 (0%)	NC	NC	NC
**Microbiology results**				94/131 (71.8%)	43/54 (79.6%)	51/77 (66.2%)	0.094	1.992 (0.883–4.495)	0.096
	Sterile			6/94 (6.4%)	3/43 (7.0%)	3/51 (5.9%)	1.000	1.199 (0.229–6.275)	0.828
	Polymicrobial			47/94 (50.0%)	24/43 (55.8%)	23/51 (45.1%)	0.301	1.537 (0.679–3.478)	0.301
	*S.aureus*			36/94 (38.3%)	14/43 (32.6%)	22/51 (43.1%)	0.293	0.636 (0.273–1.481)	0.294
	CoNS			6/94 (6.4%)	3/43 (7.0%)	3/51 (5.9%)	1.000	1.199 (0.229–6.275)	0.828
	*Streptococcus* spp.			8/94 (8.5%)	4/43 (9.3%)	4/51 (7.8%)	1.000	1.205 (0.282–5.134)	0.800
	*Enterococcus* spp.			16/94 (17.0%)	6/43 (14.0%)	10/51 (19.6%)	0.467	0.664 (0.220–2.008)	0.469
	*Corynebacterium* spp.			3/94 (3.2%)	2/43 (4.7%)	1/51 (2.0%)	0.591	2.439 (0.213–27.863)	0.473
	*Cutibacterium* spp.			2/94 (2.1%)	2/43 (4.7%)	0/51 (0%)	0.207	NC	NC
	Enterobacteriaceae			29/94 (30.9%)	14/43 (32.6%)	15/51 (29.4%)	0.742	1.158 (0.481–2.785)	0.742
	*Pseudomonas* spp.			5/94 (5.3%)	2/43 (4.7%)	3/51 (5.9%)	1.000	0.780 (0.124-4.900)	0.791
	Anaerobes			22/94 (23.4%)	11/43 (25.6%)	11/51 (21.6%)	0.647	1.249 (0.480–3.252)	0.647
	*Actinomyces* spp.			4/94 (4.3%)	2/43 (4.7%)	2/51 (3.9%)	1.000	1.195 (0.161–8.860)	0.861
	Fungi			3/94 (3.2%)	3/43 (7.0%)	0/51 (0%)	0.092	NC	NC
**Infection management**									
	Local treatment only			45/131 (34.4%)	22/54 (40.7%)	23/77 (29.9%)	0.197	1.614 (0.777–3.349)	0.198
	Radiological drainage			18/131 (13.7%)	6/54 (11.1%)	12/77 (15.6%)	0.608	0.677 (0.237–1.932)	0.466
	Surgery			41/131 (31.3%)	17/54 (31.5%)	24/77 (31.2%)	0.970	1.014 (0.479–2.147)	0.969
		Partial mesh removal		11/131 (8.4%)	3/54 (5.6%)	8/77 (10.4%)	0.524	0.507 (0.128–2.007)	0.333
		Single-stage abdominal wall reconstruction		0/51 (0%)	0/20 (0%)	0/31 (0%)	NC	NC	NC
	Negative pressure therapy alone			4/131 (3.1%)	1/54 (1.9%)	3/77 (3.9%)	0.643	0.465 (0.047–4.598)	0.512
	Post-operative negative pressure therapy			29/131 (22.1%)	8/54 (14.8%)	21/77 (27.3%)	0.134	0.463 (0.188–1.143)	0.095
**Antibiotic treatment**				112/130 (86.2%)	42/53 (79.2%)	70/77 (90.9%)	0.058	0.381 (0.137–1.060)	0.064
	Adapted empiric antibiotic therapy			58/75 (77.3%)	22/29 (75.9%)	36/46 (78.3%)	0.809	0.873 (0.290–2.627)	0.809
	Secondary adaptation			41/68 (60.3%)	13/25 (52.0%)	28/43 (65.1%)	0.286	0.580 (0.212–1.584)	0.288
	Treatment duration (days)			14 (9–21)	11 (7–19)	14 (0–21)	0.086	0.996 (0.972–1.021)	0.782
**Outcomes**									
	Failure			54/131 (41.2%)	54/54 (100%)	NA	NA	NA	NA
	Time to failure (days)			36.5 (16–183)	36.5 (16–183)	NA	NA	NA	NA
	Hernia recurrence			32/131 (24.4%)	16/54 (29.6%)	16/77 (20.8%)	0.246	1.605 (0.719–3.582)	0.247
	Subsequent surgery for infectious purpose			42/131 (32.1%)	42/54 (77.8%)	NA	NA	NA	NA
	Follow-up time (weeks)			76 (25.9-201.1)	73.2 (32.3-180.3)	77.9 (26-203.3)	0.861	1.000 (0.997–1.002)	0.846

95%CI, 95% confidence interval; ASA, American Society of Anesthesiologists, BMI, Body mass index; CoNS, Coagulase negative staphylococci; CRP, C-reactive protein; NA, Not applicable; NC, Not calculable; OR, Odd ratio

In multivariate analysis, multiple previous abdominal surgeries (OR, 4.335; 95%CI, 1.534–12.252; *p* = 0.006), symptoms duration superior to one month (OR, 3.378; 95%CI, 1.089–10.476; *p* = 0.035), and retromuscular mesh placement (OR, 0.444; 95%CI, 0.199–0.992; *p* = 0.048) remained independent predictors for failure.

## Discussion

Our study represents one of the largest series examining characteristics, management strategies and outcome determinants of abdominal wall mesh infection [6, 8, 14]. However, some limitations must be acknowledged. First, the retrospective monocentric design of our study introduces inherent biases. Our tertiary care center tends to handle more complex cases with greater comorbidity and surgical complexity, potentially influencing treatment outcomes. Nevertheless, the diversity of practices across the four different visceral surgery wards within our center provides a comprehensive perspective. Notably, results from the subgroup analysis of patients not referred to our center (HCL group) were consistent with those of the total population, mitigating concerns regarding referral bias. A key limitation stems from the retrospective nature of patient recruitment, relying on keyword searches within medical records. This method may have overlooked minor wound events deemed insignificant by surgeons, resulting in underreporting of less severe infections such as inflammatory wound dehiscence. Consequently, our conclusions may not be generalized to cases presenting solely as wound dehiscence. Also, because of the retrospective design and the clinical definition of infection, it is difficult to state whether patients experiencing a complex healing course of the surgical wounds and who did not undergo subsequent surgical procedures had actually an infection involving the mesh. This doubt is in line with the observation that 24/146 (16.4%) patients were cured without local treatment only (i.e., wound care), arguing that they might have experienced a superficial surgical site infection or a wound hazard, but no deep infection. However, we decided to keep them in our analysis because they were considered as suspect of mesh infection by the treating team. Future works using a prospective design and standardized approach of wound management after abdominal wall repair could address this question. Nonetheless, our study offers valuable insights into the management and predictive factors for treatment success in more challenging presentations, such as abscesses and fistulas.

Microbiological analysis provided precise data, mostly consistent with the literature although the rate of *S. aureus* infections (36.1%) appeared lower than usually described (46–66%) [8, 14–16], but higher than in a recent French cohort from another tertiary care center (23%) [17]. Our findings regarding microbial distribution according to infection depth revealed a gradient in bacterial types, reflecting distinct pathophysiological mechanisms between deep infections where gut commensal bacteria are prevailing, and superficial infections where skin commensal bacteria are predominant. Regarding empiric antibiotic treatment, combinations of amoxicillin plus clavulanate, or cefotaxime plus metronidazole, represent good options as they offer coverage regarding most strains of *S. aureus*,* Enterobacteriaceae*, and streptococci. Empiric use of broad-spectrum antimicrobials is not warranted in our setting. While differences in bacterial distribution were observed, it seems appropriate to use the same antibiotics for these different infection locations and types. Finally, pre-surgical pneumoperitoneum was associated with higher occurrence of MDR bacterial infection. As this technic is usually proposed for the most complicated abdominal wall repairs, pre-surgical invasive procedure and prolonged hospital stay in more comorbid patients might facilitate surgical site colonization and infection by nosocomial flora.

Management of hernia mesh infection is not codified and remains challenging, as evidenced by the 30.1% treatment failure rate in our study. This rate is congruent with 32.5% (39/120) patients presenting an infection after treatment in the cohort of Zou et al. [8]. Our study confirms the crucial impact of complete mesh removal for infection resolution, while partial mesh removal showed no significant impact on outcomes. Antibiotic therapy appears to be the second cornerstone of hernia mesh infection management.

In cases where complete surgical removal poses significant risks or challenges, conservative treatment may be considered. However, success rate appears to be lower, particularly in patients with chronic infection (symptom duration > 1 month), non-retromuscular mesh placement, and a history of multiple surgeries. These factors exerted a cumulative effect on treatment success, underscoring the importance of careful patient selection and stratification before adopting a conservative strategy.

While previous studies have highlighted certain predictors of treatment outcomes in conservative strategies, such as the early onset of infection [18], this finding regarding symptom duration as a prognostic factor is original. The association between symptom duration and treatment failure aligns with biofilm-related complications observed in orthopedic prosthetic material infections and the existing proof regarding biofilm development on hernia mesh [19, 20].

In conclusion, hernia mesh infection management remains challenging with high risk of treatment failure. The strategy of combining complete mesh removal with antibiotic therapy is the most promising approach for achieving successful outcomes. When conservative treatment is being considered, the physician must evaluate symptom duration and mesh placement, as the association of symptom duration > 1 month with non retromuscular located mesh in a multi operated abdomen is associated with > 75% failure risk.
